# Supplementary material for: Persistent equatorial Pacific iron limitation under ENSO forcing
Source: Nature. 2023 Aug 16;621(7978):330–5. doi: 10.1038/s41586-023-06439-0 (PMC10499608; doi:10.1038/s41586-023-06439-0)
Supplement: Supplementary file 1 — This file contains Supplementary Discussion 1 (discussion related to controls on diel cycles of Fv/Fm and σPSII in Fe-limited waters), Tables 1–3 (tables related to the metaproteomic analyses methods), Figs. 1 (figure exemplifying the flow cytometry gating strategy) and 2 (figure showing the performance of shipboard radiometry determination of chlorophyll a concentrations) and References. [file 41586_2023_6439_MOESM1_ESM.pdf]

nature portfolio

https://doi.org/10.1038/s41586-023-06439-0

Supplementary information

Persistent equatorial Pacific iron limitation under ENSO forcing

In the format provided by the
authors and unedited

Supplementary Information for 'Persistent Equatorial Pacific iron limitation under ENSO forcing' by T.J. Browning et al.

Supplementary Discussion 1. Controls on diel cycles of F_v/F_m and σ_{PSII} in Fe limited waters

Large nocturnal reductions in F_v/F_m and σ_{PSII} characterized the Fe limited portion of our transect, matching earlier findings (Fig. 2a,b)[10,24,25]. Proposed mechanisms for the night-time decreases in F_v/F_m are night-time reduction of the acceptor side of PSII via a back-transfer of electrons from the PQ pool, reducing F_v[10,32], and/or night-time pigment decoupling from PSII increasing background fluorescence[10,24]. To further pin down the drivers, we extracted a diurnal cycle of these measurements from the Fe limited part of the transect (boxed region in 2a,b). For these data we find that reductions in F_v/F_m are driven by both reductions in F_v and increases in F_m, with around 50% contributions from each (Fig. 2e,g).

Furthermore, the majority of the variability in F_v was found to be related to the co-occurring variability in σ_{PSII}. From first principles we have[78]:

$$F_o \propto \frac{k_f}{k_p + k_f + k_d} \sigma_{LHII}[RCII]$$

and:

$$F_m \propto \frac{k_f}{k_f + k_d} \sigma_{LHII}[RCII]$$

Where F_o and F_m are the minimum and maximum fluorescence values measured, k_f, k_p and k_d are the intrinsic rate constants for fluorescence, photochemistry and non-radiative decay respectively, [RCII] is the concentration of RCIIs within the measured volume and σ_{LHII} is the average absorption cross section of the light harvesting system of all the RCII.

Further taking[78,79]:

$$\sigma_{PSII} = \frac{k_p}{k_p + k_f + k_d} \sigma_{LHII}$$

where σ_{PSII} is absorption cross of PSII photochemistry (as measured using a single turnover active chlorophyll fluorescence technique such as Fast Repetition Rate fluorometry), we have:

$$F_o \propto \frac{k_f}{k_p} \sigma_{PSII}[RCII]$$

and

$$F_m \propto \frac{k_f(k_p + k_f + k_d)}{(k_f + k_d)(k_p + k_f + k_d)} \sigma_{PSII}[RCII]$$

Thus:

$$F_v = F_m - F_o \propto \frac{1}{1 + {k_d}/{k_f}} \sigma_{PSII}[RCII]$$

And therefore:

$$\frac{F_v}{\sigma_{PSII}} \propto \frac{1}{1 + {k_d}/{k_f}} [RCII]$$

Indeed, when F_v is normalized to σ_{PSII} (i.e., considering F_v/σ_{PSII}) we find no clear night-time reduction (Fig. 2f). This implies that the night-time F_v reductions are largely driven by the accompanying σ_{PSII} reductions (Fig. 2b) as a result of less excitation energy delivery to PSII. This lower excitation transfer to PSII in turn likely results from decoupling of light harvesting antenna at night[24,29]. The limited changes in F_v/σ_{PSII} thus argues against a role for electron back-transfers from a more highly reduced PQ pool to PSII in causing the night-time F_v reductions[10,32] as this would lower F_v independent of σ_{PSII}.

The remaining contribution to night-time F_v/F_m decreases comes from F_m increases (Fig. 2g). For this to be driven by a night-time decoupling of pigments from PSII[24] requires that the uncoupled chlorophyll pigment is more fluorescent than chlorophyll in a closed PSII reaction centre[33]. Noting that PSI does not contribute significantly to fluorescence at physiological temperatures[80], the most likely explanation for the increase in F_m is therefore energetic decoupling of additional pigment from PSI. In contrast to antenna decoupling from PSII[10,29], only a small amount of pigment decoupling from PSI could lead to a large increase in background fluorescence, because any chlorophyll will have a fluorescence yield of near zero when attached to PSI and much higher when detached[80]. Relatively enhanced PSI-associated pigments under Fe limited conditions could in turn be related to maximizing daytime energy supply to a greatly diminished pool of PSI (Fig. 2d)[30,31]. Correspondingly, the detachment of antenna from both PSII and PSI may correspond to a more reduced state of the night-time PQ pool under Fe limiting conditions associated with the lower cytochrome-b_6f concentrations (Fig. 2) restricting respiratory electron flow from the NAD(P)H dehydrogenase, through the PQ pool then the cytochrome b_6f complex and on to the cytochrome c oxidase/plastocyanin[32,81].

Supplementary Tables

Table S1. Proteins extracted from the metaproteomic dataset and used in Figures 1d and 2d. ESC indicates total exclusive spectral counts.

Site	Peptide sequence	ESC	Lowest common ancestor
Protein: Node ID:	urea ABC transporter substrate-binding protein [Prochlorococcus marinus] NODE_56034_length_1438_cov_5.52567_18_1295_-		
1	SPAAGGDFFLVGSDYVFPR	1	Prochlorococcus
2	QAVEDAGTFDDNAVR	2	Prochlorococcus
2	SPAAGGDFFLVGSDYVFPR	2	Prochlorococcus
3	QAVEDAGTFDDNAVR	2	Prochlorococcus
3	SPAAGGDFFLVGSDYVFPR	2	Prochlorococcus
4	ALPEGGIIINTLNGDQNVAFFK	203	Prochlorococcus
4	DAFLYYPIQYEAQECSNNIFYTGATPNQQSEPATDFMYK	203	Prochlorococcus
4	FDAPQGPVEVMPNHHLSQTVR	203	Prochlorococcus
4	GFACDWTDPSK	203	Synechococcales
4	GFACDWTDPSKGEK	203	Synechococcales
4	IEYIVEDGASDWPTFAEK	203	Synechococcales
4	IGEINAEGGFTILEETGVVLPQAWNQK	203	Prochlorococcus
4	IGEINAEGGFTILEETGVVLPQAWNQKHPSSK	203	Prochlorococcus
4	KALPEGGIIINTLNGDQNVAFFK	203	Prochlorococcus
4	KAMLPVYESK	203	Synechococcales
4	KLIDQDGVPVVFGGWTSASR	203	Prochlorococcus
4	LIDQDGVPVVFGGWTSASR	203	Prochlorococcus
4	LIDQDGVPVVFGGWTSASRK	203	Prochlorococcus
4	MAIEEINAAGGVK	203	Prochlorococcus
4	QAVEDAGTFDDNAVR	203	Prochlorococcus
4	QLGGKVVGEDYLPLGNTEVAPIISK	203	Prochlorococcus
4	RSPAAGGDFFLVGSDYVFPR	203	Prochlorococcus
4	SPAAGGDFFLVGSDYVFPR	203	Prochlorococcus
4	SYKIEYIVEDGASDWPTFAEK	203	Synechococcales
4	VVADPQESAYNMVYLWK	203	Synechococcales
4	VVGEDYLPLGNTEVAPIISK	203	Prochlorococcus
4	VVGEDYLPLGNTEVAPIISKIK	203	Prochlorococcus
4	WGADRVVADPQESAYNMVYLWK	203	Synechococcales
5	ALPEGGIIINTLNGDQNVAFFK	72	Prochlorococcus
5	DAFLYYPIQYEAQECSNNIFYTGATPNQQSEPATDFMYK	72	Prochlorococcus
5	FDAPQGPVEVMPNHHLSQTVR	72	Prochlorococcus
5	GFACDWTDPSK	72	Synechococcales
5	GFACDWTDPSKGEK	72	Synechococcales

5	IEYIVEDGASDWPTFAEK	72	Synechococcales
5	IGEINAEGGFTILEETGVVLPQAWNQK	72	Prochlorococcus
5	KLIDQDGVPVVFGGWTSASR	72	Prochlorococcus
5	LIDQDGVPVVFGGWTSASR	72	Prochlorococcus
5	MAIEEINAAGGVK	72	Prochlorococcus
5	QAVEDAGTFDDNAVR	72	Prochlorococcus
5	QLGGKVVGEDYLPLGNTEVAPIISK	72	Prochlorococcus
5	SPAAGGDFFLVGSDYVFPR	72	Prochlorococcus
5	VVADPQESAYNMVYLWK	72	Synechococcales
5	VVGEDYLPLGNTEVAPIISK	72	Prochlorococcus
Protein:	MULTISPECIES: P-II family nitrogen regulator [Prochlorococcus]		
Node ID:	NODE_385576_length_504_cov_2.59911_61_399_+		
1	GSEFTVEFLQK	1	Synechococcales
2	GSEFTVEFLQK	1	Synechococcales
3	GSEFTVEFLQK	1	Synechococcales
4	GSEFTVEFLQK	14	Synechococcales
4	IALVNSGIVGMTVSEVR	14	Prochlorococcus
4	IFITSIDSVVR	14	Prochlorococcus
4	VEVVVEDEKVNSVIDAIAEAAK	14	Prochlorococcus
4	VNSVIDAIAEAAK	14	Prochlorococcus
5	GSEFTVEFLQK	8	Synechococcales
5	IALVNSGIVGMTVSEVR	8	Prochlorococcus
5	VEVVVEDEKVNSVIDAIAEAAK	8	Prochlorococcus
5	VNSVIDAIAEAAK	8	Prochlorococcus
Protein:	global nitrogen regulator NtcA [Prochlorococcus marinus]		
Node ID:	NODE_1088450_length_331_cov_1.03261_1_331_+		
4	AIEEDASVGLLLLQGLSSR	4	Prochlorococcus
4	ILQTETMIETLTHR	4	Synechococcales
4	LSHQAIAEAIGSTR	4	Cyanobacteria
5	AIEEDASVGLLLLQGLSSR	3	Prochlorococcus
5	ILQTETMIETLTHR	3	Synechococcales
Protein:	flavodoxin FldA [Prochlorococcus marinus]		
Node ID:	NODE_65185_length_1202_cov_3.01656_746_1202_-		
1	AGADMVGYVDK	25	Prochlorococcus
1	LETWASQLKGEIPSLG	25	Prochlorococcus
1	SGTAWDSILEDIGELSLSGK	25	Prochlorococcus
1	SGTAWDSILEDIGELSLSGKK	25	Prochlorococcus
1	SIIGESFCGLPLDEDSESDLTDSR	25	Prochlorococcus
1	SSYTFEESK	25	
1	VAIFGLGDSSTYTENYCDAMEELHSYFQK	25	
2	AGADMVGYVDK	43	Prochlorococcus

2	DVSDVDDLSELEGLDGIICGIPTWNTGADEER	43	Prochlorococcus
2	KVAIFGLGDSSTYTENYCDAMEELHSYFQK	43	
2	LETWASQLKGEIPSLG	43	Prochlorococcus
2	SGTAWDSILEDIGELSLSGK	43	Prochlorococcus
2	SGTAWDSILEDIGELSLSGKK	43	Prochlorococcus
2	SIIGESFCGLPLDEDSESDLTDSR	43	Prochlorococcus
2	SSYTFEESK	43	
2	VAIFGLGDSSTYTENYCDAMEELHSYFQK	43	
3	AGADMVGYVDK	38	Prochlorococcus
3	AGADMVGYVDKSSYTFEESK	38	Prochlorococcus
3	LETWASQLKGEIPSLG	38	Prochlorococcus
3	SGTAWDSILEDIGELSLSGK	38	Prochlorococcus
3	SGTAWDSILEDIGELSLSGKK	38	Prochlorococcus
3	SIIGESFCGLPLDEDSESDLTDSR	38	Prochlorococcus
3	VAIFGLGDSSTYTENYCDAMEELHSYFQK	38	
4	AGADMVGYVDK	15	Prochlorococcus
4	SGTAWDSILEDIGELSLSGK	15	Prochlorococcus
4	SGTAWDSILEDIGELSLSGKK	15	Prochlorococcus
4	VAIFGLGDSSTYTENYCDAMEELHSYFQK	15	
5	AGADMVGYVDK	21	Prochlorococcus
5	DVSDVDDLSELEGLDGIICGIPTWNTGADEER	21	Prochlorococcus
5	SGTAWDSILEDIGELSLSGK	21	Prochlorococcus
5	SGTAWDSILEDIGELSLSGKK	21	Prochlorococcus
5	SIIGESFCGLPLDEDSESDLTDSR	21	Prochlorococcus
Protein:	putative aminotransferase [Prochlorococcus marinus str. MIT 9215]		
Node ID:	NODE_198520_length_669_cov_1.64658_1_669_+		
4	GAEAVYSLEGK	3	Prochlorococcus
4	GLSSTNEEIDLWPLWNR	3	Prochlorococcus
4	VSVVGTPGSGFGLSGEGYFR	3	Prochlorococcus
5	GAEAVYSLEGK	6	Prochlorococcus
5	GLSSTNEEIDLWPLWNR	6	Prochlorococcus
5	VSVVGTPGSGFGLSGEGYFR	6	Prochlorococcus
5	VYGGDNAPYIWIK	6	Prochlorococcus
Protein:	photosystem II chlorophyll-binding protein CP47 [Prochlorococcus marinus]		
Node ID:	NODE_71998_length_1138_cov_2.28163_1_994_+		
1	AQLGEAFKFDR	26	Prochlorococcus
1	IPNFFENFPVILEDK	26	Prochlorococcus
1	IPNFFENFPVILEDKEGNVR	26	Prochlorococcus
1	LAFYDYVGNSPAK	26	Unclassified_Cyanobacteria
1	RIPNFFENFPVILEDK	26	Prochlorococcus
1	VGALVNGDGLPTGWQGHISFQDK	26	Prochlorococcus

1	VGALVNGDGLPTGWQGHISFQDKEGNELEVR	26	Prochlorococcus
1	VQTAIDNGATKEEAYASIPEK	26	Prochlorococcus
1	YSFEQTGITATIYGGDLNGQTFTDPAVVKR	26	Prochlorococcus
2	AQLGEAFKFDR	36	Prochlorococcus
2	FAGISADFGDQVEFGLFK	36	
2	FAGISADFGDQVEFGLFKK	36	
2	IPNFFENFPVILEDK	36	Prochlorococcus
2	IPNFFENFPVILEDKEGNVR	36	Prochlorococcus
2	LAFYDYVGNSPAK	36	Unclassified_Cyanobacteria
2	RIPNFFENFPVILEDK	36	Prochlorococcus
2	VGALVNGDGLPTGWQGHISFQDK	36	Prochlorococcus
2	VGALVNGDGLPTGWQGHISFQDKEGNELEVR	36	Prochlorococcus
2	VQTAIDNGATKEEAYASIPEK	36	Prochlorococcus
2	YSFEQTGITATIYGGDLNGQTFTDPAVVK	36	Prochlorococcus
2	YSFEQTGITATIYGGDLNGQTFTDPAVVKR	36	Prochlorococcus
3	EEAYASIPEK	30	Prochlorococcus
3	FAGISADFGDQVEFGLFK	30	
3	IPNFFENFPVILEDK	30	Prochlorococcus
3	IPNFFENFPVILEDKEGNVR	30	Prochlorococcus
3	LAFYDYVGNSPAK	30	Unclassified_Cyanobacteria
3	RIPNFFENFPVILEDK	30	Prochlorococcus
3	VGALVNGDGLPTGWQGHISFQDK	30	Prochlorococcus
3	VQTAIDNGATKEEAYASIPEK	30	Prochlorococcus
3	YSFEQTGITATIYGGDLNGQTFTDPAVVK	30	Prochlorococcus
3	YSFEQTGITATIYGGDLNGQTFTDPAVVKR	30	Prochlorococcus
4	IPNFFENFPVILEDK	30	Prochlorococcus
4	IPNFFENFPVILEDKEGNVR	30	Prochlorococcus
4	LAFYDYVGNSPAK	30	Unclassified_Cyanobacteria
4	RIPNFFENFPVILEDK	30	Prochlorococcus
4	VGALVNGDGLPTGWQGHISFQDK	30	Prochlorococcus
4	VQTAIDNGATKEEAYASIPEK	30	Prochlorococcus
4	YSFEQTGITATIYGGDLNGQTFTDPAVVK	30	Prochlorococcus
4	YSFEQTGITATIYGGDLNGQTFTDPAVVKR	30	Prochlorococcus
5	IPNFFENFPVILEDK	25	Prochlorococcus
5	IPNFFENFPVILEDKEGNVR	25	Prochlorococcus
5	LAFYDYVGNSPAK	25	Unclassified_Cyanobacteria
5	RIPNFFENFPVILEDK	25	Prochlorococcus
5	VGALVNGDGLPTGWQGHISFQDK	25	Prochlorococcus
5	YSFEQTGITATIYGGDLNGQTFTDPAVVK	25	Prochlorococcus
5	YSFEQTGITATIYGGDLNGQTFTDPAVVKR	25	Prochlorococcus
Protein:	photosystem II protein D1 (PsbA) [Prochlorococcus marinus str. MIT 9116]		

Node ID:	NODE_710319_length_396_cov_5.07038_1_396_+		
1	ANLGMEVMHER	10	Cyanobacteria
1	ETTGLESQNYGYK	10	
1	FGQEEETYNIVAAHGYFGR	10	Cyanobacteria
1	IVPTWGDVLNR	10	Prochlorococcus
1	LIFQYASFNNSR	10	Cyanobacteria
2	ANLGMEVMHER	14	Cyanobacteria
2	ETTGLESQNYGYK	14	
2	FGQEEETYNIVAAHGYFGR	14	Cyanobacteria
2	IVPTWGDVLNR	14	Prochlorococcus
3	ANLGMEVMHER	8	Cyanobacteria
3	ETTGLESQNYGYK	8	
3	FGQEEETYNIVAAHGYFGR	8	Cyanobacteria
3	IVPTWGDVLNR	8	Prochlorococcus
4	ANLGMEVMHER	11	Cyanobacteria
4	ETTGLESQNYGYK	11	
4	FGQEEETYNIVAAHGYFGR	11	Cyanobacteria
4	IVPTWGDVLNR	11	Prochlorococcus
5	ANLGMEVMHER	8	Cyanobacteria
5	FGQEEETYNIVAAHGYFGR	8	Cyanobacteria
5	IVPTWGDVLNR	8	Prochlorococcus
Protein:	Photosystem II manganese-stabilizing protein [Prochlorococcus marinus str. AS9601]		
Node ID:	NODE_64088_length_1214_cov_2.04573_330_1124_+		
1	AANTGVEYAQGLVALGGDDEELAK	12	
1	AANTGVEYAQGLVALGGDDEELAKENIK	12	
1	GICMHPTEVYVK	12	Prochlorococcus
1	GNAQFTDVVNTGK	12	Prochlorococcus
1	NNTTVTEVYGDIDSGK	12	
1	NNTTVTEVYGDIDSGKFTEK	12	
2	AANTGVEYAQGLVALGGDDEELAK	16	
2	AANTGVEYAQGLVALGGDDEELAKENIK	16	
2	ANDCPEIDSSSIGSISLSNGDSLK	16	
2	DLTASIPSSIEPGTELSGTTFTPNYR	16	
2	GICMHPTEVYVK	16	Prochlorococcus
2	GNAQFTDVVNTGK	16	Prochlorococcus
2	NNTTVTEVYGDIDSGK	16	
2	NNTTVTEVYGDIDSGKFTEK	16	
3	AANTGVEYAQGLVALGGDDEELAK	18	
3	AANTGVEYAQGLVALGGDDEELAKENIK	18	
3	ANDCPEIDSSSIGSISLSNGDSLK	18	
3	DLTASIPSSIEPGTELSGTTFTPNYR	18	
3	GICMHPTEVYVK	18	Prochlorococcus

3	GNAQFTDVVNTGK	18	Prochlorococcus
3	NNTTVTEVYGDIDSGK	18	
3	NNTTVTEVYGDIDSGKFTEK	18	
4	AANTGVEYAQGLVALGGDDEELAK	6	
4	AANTGVEYAQGLVALGGDDEELAKENIK	6	
4	GICMHPTEVYVK	6	Prochlorococcus
4	GNAQFTDVVNTGK	6	Prochlorococcus
4	NNTTVTEVYGDIDSGK	6	
5	GICMHPTEVYVK	5	Prochlorococcus
5	GNAQFTDVVNTGK	5	Prochlorococcus
5	NNTTVTEVYGDIDSGK	5	
Protein:	photosystem II 44 kDa subunit reaction center protein [Prochlorococcus sp. HOT208_60m_813I02]		
Node ID:	NODE_142315_length_843_cov_4.54442_1_659_+		
1	AAEYMTHAPNASINSVGGIITEPNAVNFVNLR	11	Prochlorococcus
1	GPWLEPLRGPNGLSLEK	11	Prochlorococcus
1	IGANVGSTMGPTGLGK	11	Prochlorococcus
1	SPTGEIIFGGETMR	11	Cyanobacteria
2	AAEYMTHAPNASINSVGGIITEPNAVNFVNLR	12	Prochlorococcus
2	FWDFRGPWLEPLR	12	Unclassified_Cyanobacteria
2	IGANVGSTMGPTGLGK	12	Prochlorococcus
2	SPTGEIIFGGETMR	12	Cyanobacteria
3	AAEYMTHAPNASINSVGGIITEPNAVNFVNLR	8	Prochlorococcus
3	IGANVGSTMGPTGLGK	8	Prochlorococcus
3	SPTGEIIFGGETMR	8	Cyanobacteria
4	AAEYMTHAPNASINSVGGIITEPNAVNFVNLR	12	Prochlorococcus
4	FWDFRGPWLEPLR	12	Unclassified_Cyanobacteria
4	IGANVGSTMGPTGLGK	12	Prochlorococcus
4	SPTGEIIFGGETMR	12	Cyanobacteria
5	AAEYMTHAPNASINSVGGIITEPNAVNFVNLR	11	Prochlorococcus
5	IGANVGSTMGPTGLGK	11	Prochlorococcus
5	SPTGEIIFGGETMR	11	Cyanobacteria
Protein:	photosystem I chlorophyll a apoprotein subunit Ib (PsaB) [Prochlorococcus marinus str. MIT 9123]		
Node ID:	NODE_316846_length_556_cov_2.49701_1_556_+		
1	QILIEPVFAQFVQAAQGK	4	Prochlorococcus
4	DLGYAFPCDGPGR	13	Prochlorococcus
4	QILIEPVFAQFVQAAQGK	13	Prochlorococcus
5	DLGYAFPCDGPGR	11	Prochlorococcus
5	QILIEPVFAQFVQAAQGK	11	Prochlorococcus
Protein:	photosystem I core protein PsaA [Prochlorococcus sp. MED-G72]		

Node ID:	NODE_4166102_length_210_cov_1.22581_1_210_+		
4	EILDSQQGDPILFPAPR	4	Prochlorococcus
4	GHQGLFEFMAESR	4	Prochlorococcus
5	EILDSQQGDPILFPAPR	4	Prochlorococcus
Protein:	MULTISPECIES: Photosystem I reaction center subunit III [Prochlorococcus]		
Node ID:	NODE_1314551_length_311_cov_1.22266_1_311_-		
4	ASCGDDGLPHLIIGPPLEPWGALLNR	4	Prochlorococcus
4	ASTEYTASALTK	4	Prochlorococcus
5	ASCGDDGLPHLIIGPPLEPWGALLNR	4	Prochlorococcus
Protein:	Cytochrome b6-f complex subunit [Prochlorococcus marinus str. MIT 9321]		
Node ID:	NODE_1569197_length_287_cov_1.14655_1_287_-		
4	EIGADGSEVPLQVGAVVMLPDGFK	1	Prochlorococcus
5	EIGADGSEVPLQVGAVVMLPDGFK	5	Prochlorococcus
5	EIVFPVLSPDPSTNK	5	Prochlorococcus

Table S2. Total spectral counts for reported *Prochlorococcus* proteins. S1–S5 are Sites 1–5.

Protein	S1	S2	S3	S4	S5
**Urea ABC transporter**					
urea ABC transporter substrate-binding protein [Prochlorococcus marinus] NODE_56034_length_1438_cov_5.52567_18_1295_-	2	2	4	255	90
urea ABC transporter substrate-binding protein [Prochlorococcus sp. MED-G73] NODE_30527_length_2081_cov_5.52863_232_1512_+	3	0	7	157	52
urea ABC transporter substrate-binding protein [Prochlorococcus sp. MED-G72] NODE_469880_length_515_cov_1.02391_1_515_+	0	0	0	69	21
putative urea ABC transporter [Prochlorococcus marinus str. MIT 9302] NODE_1269325_length_329_cov_1.62409_1_329_-	0	0	0	60	0
putative urea ABC transporter [Prochlorococcus marinus str. MIT 9302] NODE_1320476_length_291_cov_1.78814_1_291_-	0	0	0	59	18
urea ABC transporter substrate-binding protein [Prochlorococcus marinus] NODE_1678703_length_267_cov_1.06604_1_267_+	0	0	0	43	0
urea ABC transporter substrate-binding protein [Prochlorococcus marinus] NODE_7139_length_5778_cov_3.62065_1_831_-	0	0	0	10	4
**P-II nitrogen regulator**					
MULTISPECIES: P-II family nitrogen regulator [Prochlorococcus] NODE_385576_length_504_cov_2.59911_61_399_+	1	1	0	14	10
P-II family nitrogen regulator [Prochlorococcus marinus] NODE_186760_length_729_cov_1.3368_254_592_+	6	3	4	0	0
**NtcA global nitrogen regulator**					
global nitrogen regulator NtcA [Prochlorococcus marinus] NODE_1088450_length_331_cov_1.03261_1_331_+	0	0	0	14	7
global nitrogen regulator NtcA [Prochlorococcus marinus] NODE_233133_length_618_cov_1.22558_1_618_+	1	3	5	5	0
**Putative aminotransferase**					
putative aminotransferase [Prochlorococcus marinus str. MIT 9215] NODE_198520_length_669_cov_1.64658_1_669_+	0	0	0	7	8
**Flavodoxin**					
flavodoxin FldA [Prochlorococcus marinus] NODE_310708_length_561_cov_1.93281_1_435_-	29	46	32	25	42
flavodoxin FldA [Prochlorococcus marinus] NODE_65185_length_1202_cov_3.01656_746_1202_-	25	47	40	17	26
flavodoxin FldA [Prochlorococcus marinus] NODE_4183932_length_216_cov_1.18012_1_216_+	10	25	17	0	0
flavodoxin FldA [Prochlorococcus marinus XMU1408] NODE_243084_length_719_cov_1.79819_197_719_+	2	4	5	0	0
**Photosystem II CP47 chlorophyll-binding protein**					
photosystem II chlorophyll-binding protein CP47 [Prochlorococcus marinus] NODE_71998_length_1138_cov_2.28163_1_994_+	29	38	40	33	31

photosystem II chlorophyll-binding protein CP47 [Prochlorococcus marinus] NODE_313070_length_559_cov_2.31746_1_559_-	22	26	35	25	24
Photosystem II CP47 protein (PsbB) [Prochlorococcus marinus str. MIT 9322] NODE_310961_length_539_cov_2.0124_203_539_-	0	0	0	3	0
**Photosystem II D1 protein**					
photosystem II protein D1 [Prochlorococcus marinus] NODE_42108_length_1733_cov_6.52563_38_1120_-	17	24	20	25	23
photosystem II D1 protein [Prochlorococcus phage P-SSM2] NODE_205967_length_784_cov_1.97942_1_458_+	0	20	0	0	0
**Photosystem II manganese-stabilizing protein**					
Photosystem II manganese-stabilizing protein [Prochlorococcus marinus str. AS9601] NODE_64088_length_1214_cov_2.04573_330_1124_+	16	22	21	13	9
Photosystem II manganese-stabilizing polypeptide [Prochlorococcus sp. MED-G72] NODE_134288_length_870_cov_3.91166_224_870_+	0	0	19	0	0
Photosystem II manganese-stabilizing protein [Prochlorococcus marinus str. AS9601] NODE_63485_length_1333_cov_3.47418_242_1081_-	0	0	6	22	23
photosystem II manganese-stabilizing polypeptide [Prochlorococcus marinus] NODE_257680_length_589_cov_2.25094_1_589_+	0	0	0	21	20
Photosystem II manganese-stabilizing protein [Prochlorococcus marinus str. AS9601] NODE_166990_length_813_cov_1.69525_1_512_-	0	14	13	0	0
Photosystem II manganese-stabilizing protein [Prochlorococcus marinus str. AS9601] NODE_401769_length_478_cov_3.1182_1_478_-	0	0	12	0	0
photosystem II manganese-stabilizing polypeptide [Prochlorococcus sp. MIT 0604] NODE_615634_length_433_cov_1.22487_1_433_+	0	0	0	15	17
Photosystem II manganese-stabilizing protein [Prochlorococcus marinus str. MIT 9215] NODE_370447_length_514_cov_3.20915_131_514_-	10	10	14	5	1
Photosystem II manganese-stabilizing polypeptide [Prochlorococcus marinus XMU1408] NODE_2121108_length_258_cov_0.935961_1_258_+	0	2	3	0	0
**Photosystem II 44 kDa subunit reaction center protein**					
photosystem II 44 kDa subunit reaction center protein [Prochlorococcus sp. HOT208_60m_813I02] NODE_142315_length_843_cov_4.54442_1_659_+	15	15	14	17	14
MULTISPECIES: photosystem II 44 kDa subunit reaction center protein [Prochlorococcus] NODE_14192_length_3753_cov_4.27772_1180_2427_+	7	0	0	0	0
MULTISPECIES: photosystem II 44 kDa subunit reaction center protein [Prochlorococcus] NODE_3042960_length_231_cov_1.07955_1_231_+	5	5	5	2	1
**Photosystem I chlorophyll a protein subunit Ib**					
photosystem I chlorophyll a apoprotein subunit Ib (PsaB) [Prochlorococcus marinus str. MIT 9123] NODE_316846_length_556_cov_2.49701_1_556_+	3	0	0	17	15
photosystem I core protein PsaB [Prochlorococcus marinus] NODE_101709_length_941_cov_3.33183_1_796_+	0	0	0	10	6
MULTISPECIES: photosystem I core protein PsaB [Prochlorococcus] NODE_25525_length_2602_cov_3.3243_19_2262_-	0	0	0	5	3

photosystem I chlorophyll a apoprotein subunit Ib (PsaB) [Prochlorococcus sp. MIT 0601] NODE_185661_length_828_cov_1.29107_1_828_-	0	0	0	3	0
**Photosystem I reaction center subunit III**					
MULTISPECIES: Photosystem I reaction center subunit III [Prochlorococcus] NODE_144854_length_835_cov_3.49872_350_835_+	0	0	0	5	4
**Photosystem I core protein PsaA**					
photosystem I core protein PsaA [Prochlorococcus sp. MED-G72] NODE_4166102_length_210_cov_1.22581_1_210_+	0	0	0	5	5
**Cytochrome b6-f complex**					
Cytochrome b6-f complex subunit [Prochlorococcus marinus str. MIT 9321] NODE_1569197_length_287_cov_1.14655_1_287_-	0	0	0	4	5

Table S3. *Prochlorococcus* strain matches for PSI and cytochrome b_6-f peptides.

Genome Name	NCBI ID	IMG ID	Isolation location
Protein:	photosystem I chlorophyll a apoprotein subunit Ib (PsaB) [Prochlorococcus marinus str. MIT 9123]		
Node ID:	NODE_316846_length_556_cov_2.49701_1_556_+		
Sequence:	QILIEPVFAQFVQAAQGK		
Prochlorococcus sp. MIT9202	93058	647533199	South Pacific
Prochlorococcus sp. MIT9215	93060	640753041	Equatorial Pacific
Prochlorococcus sp. MIT9301	167546	640069322	Sargasso Sea (BATS)
Prochlorococcus sp. MIT9302	74545	2606217691	Sargasso Sea
Prochlorococcus sp. MIT9311	167547	2606217680	Gulf Stream
Prochlorococcus marinus MIT9312	74546	637000210	Gulf Stream
Prochlorococcus sp. MIT9314	167548	2606217312	Gulf Stream
Prochlorococcus sp. MIT9321	167549	2606217683	Equatorial Pacific
Prochlorococcus sp. MIT9322	167550	2606217679	Equatorial Pacific
Prochlorococcus sp. MIT9401	167551	2606217316	Sargasso Sea
Prochlorococcus sp. MIT9515	167542	640069324	Equatorial Pacific
Prochlorococcus sp. SB	59926	2606217677	Western Subtropical Pacific
Prochlorococcus sp. MIT9201	93057	2606217687	Tropical Pacific
Sequence:	DLGYAFPCDGPGR		
Prochlorococcus sp. MIT9202	93058	647533199	South Pacific
Prochlorococcus sp. MIT9215	93060	640753041	Equatorial Pacific
Prochlorococcus sp. MIT9301	167546	640069322	Sargasso Sea (BATS)
Prochlorococcus sp. MIT9302	74545	2606217691	Sargasso Sea
Prochlorococcus sp. MIT9311	167547	2606217680	Gulf Stream
Prochlorococcus marinus MIT9312	74546	637000210	Gulf Stream
Prochlorococcus sp. MIT9314	167548	2606217312	Gulf Stream
Prochlorococcus sp. MIT9321	167549	2606217683	Equatorial Pacific
Prochlorococcus sp. MIT9322	167550	2606217679	Equatorial Pacific
Prochlorococcus sp. MIT9401	167551	2606217316	Sargasso Sea
Prochlorococcus sp. MIT9515	167542	640069324	Equatorial Pacific
Prochlorococcus sp. SB	59926	2606217677	Western Subtropical Pacific
Prochlorococcus sp. MIT9201	93057	2606217687	Tropical Pacific
Protein:	photosystem I core protein PsaA [Prochlorococcus sp. MED-G72]		
Node ID:	NODE_4166102_length_210_cov_1.22581_1_210_+		
Sequence:	EILDSQQGDPILFPAPR		
Prochlorococcus sp. MIT9321	167549	2606217683	Equatorial Pacific
Prochlorococcus sp. MIT9322	167550	2606217679	Equatorial Pacific
Prochlorococcus sp. MIT9401	167551	2606217316	Sargasso Sea
Sequence:	GHQGLFEFMAESR		
Prochlorococcus sp. MIT9215	93060	640753041	Equatorial Pacific
Prochlorococcus sp. MIT9301	167546	640069322	Sargasso Sea (BATS)

Prochlorococcus sp. MIT9302	74545	2606217691	Sargasso Sea
Prochlorococcus sp. MIT9311	167547	2606217680	Gulf Stream
Prochlorococcus marinus MIT9312	74546	637000210	Gulf Stream
Prochlorococcus sp. MIT9314	167548	2606217312	Gulf Stream
Prochlorococcus sp. MIT9321	167549	2606217683	Equatorial Pacific
Prochlorococcus sp. MIT9322	167550	2606217679	Equatorial Pacific
Prochlorococcus sp. MIT9401	167551	2606217316	Sargasso Sea
Prochlorococcus sp. SB	59926	2606217677	Western Subtropical Pacific
Protein:	MULTISPECIES: Photosystem I reaction center subunit III [Prochlorococcus]		
Node ID:	NODE_1314551_length_311_cov_1.22266_1_311_-		
Sequence:	ASCGDDGLPHLIIGPPLEPWGALLNR		
Prochlorococcus sp. MIT9301	167546	640069322	Sargasso Sea (BATS)
Prochlorococcus sp. MIT9314	167548	2606217312	Gulf Stream
Prochlorococcus sp. MIT9321	167549	2606217683	Equatorial Pacific
Prochlorococcus sp. MIT9322	167550	2606217679	Equatorial Pacific
Prochlorococcus sp. MIT9401	167551	2606217316	Sargasso Sea
Prochlorococcus sp. SB	59926	2606217677	Western Subtropical Pacific
Prochlorococcus sp. MIT9201	93057	2606217687	Tropical Pacific
Sequence:	ASTEYTASALTK		
Prochlorococcus sp. MIT9202	93058	647533199	South Pacific
Prochlorococcus sp. MIT9215	93060	640753041	Equatorial Pacific
Prochlorococcus sp. MIT9301	167546	640069322	Sargasso Sea (BATS)
Prochlorococcus sp. MIT9314	167548	2606217312	Gulf Stream
Prochlorococcus sp. MIT9321	167549	2606217683	Equatorial Pacific
Prochlorococcus sp. MIT9322	167550	2606217679	Equatorial Pacific
Prochlorococcus sp. MIT9401	167551	2606217316	Sargasso Sea
Prochlorococcus sp. MIT9201	93057	2606217687	Tropical Pacific
Protein:	Cytochrome b6-f complex subunit [Prochlorococcus marinus str. MIT 9321]		
Node ID:	NODE_1569197_length_287_cov_1.14655_1_287_-		
Sequence:	EIGADGSEVPLQVGAVVMLPDGFK		
Prochlorococcus sp. MIT9202	93058	647533199	South Pacific
Prochlorococcus sp. MIT9215	93060	640753041	Equatorial Pacific
Prochlorococcus sp. MIT9301	167546	640069322	Sargasso Sea (BATS)
Prochlorococcus sp. MIT9311	167547	2606217680	Gulf Stream
Prochlorococcus marinus MIT9312	74546	637000210	Gulf Stream
Prochlorococcus sp. MIT9314	167548	2606217312	Gulf Stream
Prochlorococcus sp. MIT9321	167549	2606217683	Equatorial Pacific
Prochlorococcus sp. MIT9322	167550	2606217679	Equatorial Pacific
Prochlorococcus sp. MIT9401	167551	2606217316	Sargasso Sea
Prochlorococcus sp. MIT9515	167542	640069324	Equatorial Pacific
Prochlorococcus sp. SB	59926	2606217677	Western Subtropical Pacific
Prochlorococcus sp. MIT9201	93057	2606217687	Tropical Pacific

Sequence:	EIVFPVLSPDPSTNK		
Prochlorococcus sp. MIT9215	93060	640753041	Equatorial Pacific
Prochlorococcus sp. MIT9301	167546	640069322	Sargasso Sea (BATS)
Prochlorococcus sp. MIT9302	74545	2606217691	Sargasso Sea
Prochlorococcus sp. MIT9311	167547	2606217680	Gulf Stream
Prochlorococcus marinus MIT9312	74546	637000210	Gulf Stream
Prochlorococcus sp. MIT9314	167548	2606217312	Gulf Stream
Prochlorococcus sp. MIT9321	167549	2606217683	Equatorial Pacific
Prochlorococcus sp. MIT9322	167550	2606217679	Equatorial Pacific
Prochlorococcus sp. MIT9401	167551	2606217316	Sargasso Sea
Prochlorococcus sp. SB	59926	2606217677	Western Subtropical Pacific
Prochlorococcus sp. MIT9201	93057	2606217687	Tropical Pacific

Supplementary Figures

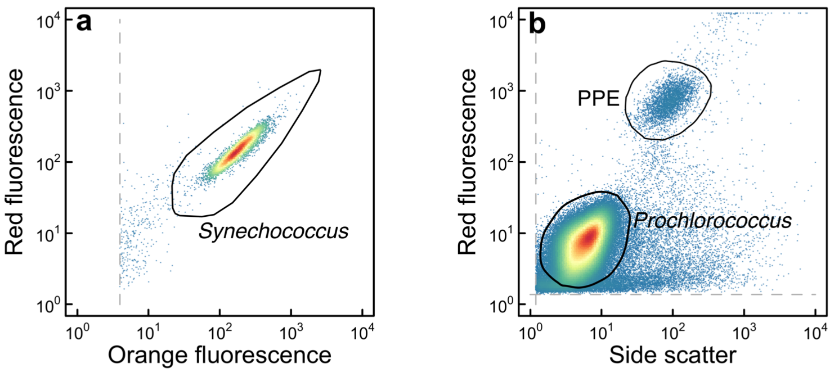

Figure S1. Example flow cytometry dot plots with gates. a, *Synechococcus* **b,** *Prochlorococcus* and photosynthetic picoeukaryotes (PPE). *Synechococcus* cells identified via their orange fluorescence in **a** have been gated out from **b**. Colours represent cell numbers (blue=low, red=high). The units for the axes are arbitrary. Grey dashed lines indicate minimum fluorescence/scatter thresholds applied for clearer visualisation in the plots.

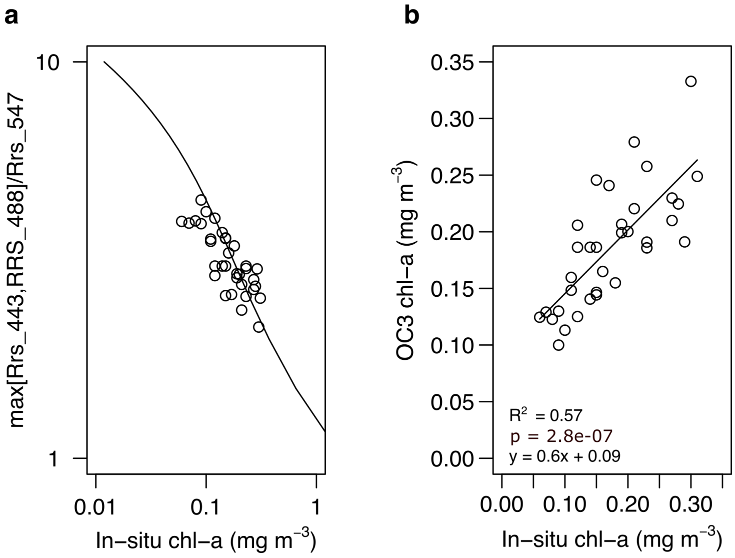

Figure S2. Performance of shipboard radiometry determination of chlorophyll-a concentrations. a, Relationship between measured in-situ chlorophyll-a concentrations and R_{RS} ratio values measured by the shipboard radiometry. The overplotted line is the R_{RS} ratio employed by the satellite OC3 algorithm. **b,** Correlation between measured in-situ chlorophyll-a concentrations and those predicted by shipboard remote sensing using the OC3 algorithm.

References for Supplementary Information (continued from main manuscript)

78. Oxborough, K., Moore, C.M., Suggett, D.J., Lawson, T., Chan, H.G. and Geider, R.J., 2012. Direct estimation of functional PSII reaction center concentration and PSII electron flux on a volume basis: a new approach to the analysis of Fast Repetition Rate fluorometry (FRRf) data. *Limnol. Oceanogr. Meth.* **10**, 142-154.
79. Kolber, Z.S., Prášil, O. and Falkowski, P.G., 1998. Measurements of variable chlorophyll fluorescence using fast repetition rate techniques: defining methodology and experimental protocols. *Biochim. Biophys. Acta - Bioenerg.* **1367**, 88-106.
80. Krause, G.H. and Weis, E., 1991. Chlorophyll fluorescence and photosynthesis: the basics. *Annu. Rev. Plant Biol.* **42**, 313-349.
81. Lea-Smith, D.J., Bombelli, P., Vasudevan, R. and Howe, C.J., 2016. Photosynthetic, respiratory and extracellular electron transport pathways in cyanobacteria. *Biochim. Biophys. Acta* **1857**, 247-255.
