# Supplementary material for: Persistent equatorial Pacific iron limitation under ENSO forcing
Source: Nature. 2023 Aug 16;621(7978):330–5. doi: 10.1038/s41586-023-06439-0 (PMC10499608; doi:10.1038/s41586-023-06439-0)
Supplement: Supplementary file 2 — Reporting Summary [file 41586_2023_6439_MOESM2_ESM.pdf]

nature portfolio

Corresponding author(s): Thomas J. Browning

Last updated by author(s): Jun 30, 2023

Reporting Summary

Nature Portfolio wishes to improve the reproducibility of the work that we publish. This form provides structure for consistency and transparency in reporting. For further information on Nature Portfolio policies, see our Editorial Policies and the Editorial Policy Checklist.

Statistics

For all statistical analyses, confirm that the following items are present in the figure legend, table legend, main text, or Methods section.

n/a	Confirmed	
☐	☒	The exact sample size (*n*) for each experimental group/condition, given as a discrete number and unit of measurement
☐	☒	A statement on whether measurements were taken from distinct samples or whether the same sample was measured repeatedly
☐	☒	The statistical test(s) used AND whether they are one- or two-sided<br>*Only common tests should be described solely by name; describe more complex techniques in the Methods section.*
☒	☐	A description of all covariates tested
☒	☐	A description of any assumptions or corrections, such as tests of normality and adjustment for multiple comparisons
☐	☒	A full description of the statistical parameters including central tendency (e.g. means) or other basic estimates (e.g. regression coefficient) AND variation (e.g. standard deviation) or associated estimates of uncertainty (e.g. confidence intervals)
☐	☒	For null hypothesis testing, the test statistic (e.g. *F*, *t*, *r*) with confidence intervals, effect sizes, degrees of freedom and *P* value noted<br>*Give P values as exact values whenever suitable.*
☒	☐	For Bayesian analysis, information on the choice of priors and Markov chain Monte Carlo settings
☒	☐	For hierarchical and complex designs, identification of the appropriate level for tests and full reporting of outcomes
☒	☐	Estimates of effect sizes (e.g. Cohen's *d*, Pearson's *r*), indicating how they were calculated

Our web collection on statistics for biologists contains articles on many of the points above.

Software and code

Policy information about availability of computer code

Data collection	Flow cytometry: CellQuest Pro v5 software (Becton Dickenson).<br>High performance liquid chromatography: Chromeleon v7.0 (Thermo Fisher Scientific).<br>Fast repetition rate florometry: FastPro8 (Chelsea Technologies).<br>ICP-MS: ELEMENT 2/XR software (Thermo Scientific)<br>Metaproteomics: Thermo Proteome Discoverer v2.2 software, Scaffold v5.0 (Proteome Software Inc.), METATRYP v2.0<br>Statistics, calculations, figures: R v4.1.0
Data analysis	Statistics and other calculations were conducted using R v4.1.0.

For manuscripts utilizing custom algorithms or software that are central to the research but not yet described in published literature, software must be made available to editors and reviewers. We strongly encourage code deposition in a community repository (e.g. GitHub). See the Nature Portfolio guidelines for submitting code & software for further information.

Data

Policy information about availability of data

All manuscripts must include a data availability statement. This statement should provide the following information, where applicable:

- Accession codes, unique identifiers, or web links for publicly available datasets
- A description of any restrictions on data availability
- For clinical datasets or third party data, please ensure that the statement adheres to our policy

Biogeochemical data are deposited in Zenodo (https://doi.org/10.5281/zenodo.8059552). Metaproteomics data are available via the PRoteomics IDEntifications (PRIDE) database (accession number: PXD030610) and ProteomeXchange (accession number: PXD030610). Hyperspectral radiometry data are available via Pangaea (https://doi.org/10.1594/PANGAEA.924038). The MODIS satellite data are available from the NASA Ocean Colour website (https://oceancolor.gsfc.nasa.gov) with the data product names 'Fluorescence Line Height (normalized), 'Chlorophyll concentration', and 'Sea Surface Temperature'.

Research involving human participants, their data, or biological material

Policy information about studies with human participants or human data. See also policy information about sex, gender (identity/presentation), and sexual orientation and race, ethnicity and racism.

Reporting on sex and gender	NA
Reporting on race, ethnicity, or other socially relevant groupings	NA
Population characteristics	NA
Recruitment	NA
Ethics oversight	NA

Note that full information on the approval of the study protocol must also be provided in the manuscript.

Field-specific reporting

Please select the one below that is the best fit for your research. If you are not sure, read the appropriate sections before making your selection.

☐ Life sciences ☐ Behavioural & social sciences ☒ Ecological, evolutionary & environmental sciences

For a reference copy of the document with all sections, see nature.com/documents/nr-reporting-summary-flat.pdf

Ecological, evolutionary & environmental sciences study design

All studies must disclose on these points even when the disclosure is negative.

Study description	Field sampling and experiments were conducted onboard the RV Sonne in January/February 2019 (SO267/2).<br>Experimental: Seawater was collected under trace-metal-clean conditions using a towed water sampling device (~2 m depth) and filled in 1 L acid-washed polycarbonate bottles (Nalgene). Triplicate amendments of nutrients (see Methods section for full details) were performed and were incubated for 2 days. Additionally, three bottles were incubated with no amendment (controls) and three were sampled for initial conditions. Nutrient, trace element and phytoplankton community structure samples were collected alongside experimental seawater. Following incubation, bottles were sub-sampled for chlorophyll-a concentrations, flow cytometry cell counts, fast repetition rate fluorometry and diagnostic pigments (pooled samples from triplicate replicates).<br>Underway sampling: Seawater was collected under trace-metal-clean conditions using a towed water sampling device (~2 m depth) for macronutrients, dissolved iron, chlorophyll-a concentrations, flow cytometry cell counts, fast repetition rate fluorometry, diagnostic pigments, and metaproteomics (5 sites only). Radiometric quantities were recorded continuously via hyperspectral radiometers at the bow of the ship.
Research sample	Natural mixed assemblages of microbial communities in surface seawaters encountered on the research cruise.
Sampling strategy	Nutrient amendment experiments were conducted with triplicate biological replicates, thus allowing for statistical testing whilst remaining logistically feasible in carrying out the field study. The underway sampling and nutrient amendment experiments were conducted at the highest spatial and temporal resolution possible during the oceanographic research cruise.
Data collection	Samples were collected by T. Browning, X. Wang, S. Garaba, and D. Voss on the research cruise. Samples were analyzed by T. Browning and several technical staff at GEOMAR Helmholtz Centre for Ocean Research Kiel (Germany). Proteomics samples were analyzed by M. McIlvin and D. Moran at Woods Hole Oceanographic Institute (USA).
Timing and spatial scale	Samples were collected between 28th January - 14th February 2019. Underway sampling was continuous or at as high temporal

Timing and spatial scale	frequency as possible. Sampling for bioassay experiments was conducted at regular intervals, setting up a new experiment after the previous had ended. Experimental samples were collected at night time in order that phytoplankton were dark acclimated.
Data exclusions	No data excluded
Reproducibility	Experimental: Identical experiments were conducted 4 times at different locations with treatments having triplicate replication. This was the maximum reproducibility possible during the fieldwork.
Randomization	Incubation bottles for the nutrient amendment experiments were filled at random.
Blinding	Investigators were not blinded to nutrient treatments.

Did the study involve field work? ☒ Yes ☐ No

Field work, collection and transport

Field conditions	The seawater temperatures throughout the research cruise are shown in Extended Data Figure 1a. Sea conditions were calm.
Location	Samples were collected within the following domain: 151 degrees west to 107 degrees west, 3 degrees south to 17 degrees north. Samples were all collected from the near-sea surface (~2 m depth).
Access & import/export	No sampling was conducted within the Exclusive Economic Zones of any country.
Disturbance	Minimal disturbance was generated by the open ocean fieldwork activities (i.e., the presence of the research ship and towing of the seawater sampling device). All chemicals and seawater exposed to chemicals were transported back to Germany for disposal.

Reporting for specific materials, systems and methods

We require information from authors about some types of materials, experimental systems and methods used in many studies. Here, indicate whether each material, system or method listed is relevant to your study. If you are not sure if a list item applies to your research, read the appropriate section before selecting a response.

Materials & experimental systems

n/a	Involved in the study
☒	☐ Antibodies
☒	☐ Eukaryotic cell lines
☒	☐ Palaeontology and archaeology
☒	☐ Animals and other organisms
☒	☐ Clinical data
☒	☐ Dual use research of concern
☒	☐ Plants

Methods

n/a	Involved in the study
☒	☐ ChIP-seq
☐	☒ Flow cytometry
☒	☐ MRI-based neuroimaging

Flow Cytometry

Plots

Confirm that:

☒ The axis labels state the marker and fluorochrome used (e.g. CD4-FITC).

☒ The axis scales are clearly visible. Include numbers along axes only for bottom left plot of group (a 'group' is an analysis of identical markers).

☒ All plots are contour plots with outliers or pseudocolor plots.

☒ A numerical value for number of cells or percentage (with statistics) is provided.

Methodology

Sample preparation	Samples (2 mL) were fixed with neutralized paraformaldehyde at a 1% final concentration (paraformaldehyde: methanol-free 16% 10 mL glass ampules, Alfa Aesar/Thermo Fisher), vortex-mixed, and left in the dark for 10 minutes before being transferred to a –80 °C freezer. Samples were thawed at room temperature before analysis.
Instrument	FACSCalibur flow cytometer (Becton Dickenson, Oxford, United Kingdom).

Software	CellQuest software (Becton Dickenson).
Cell population abundance	Identification and counts of phytoplankton populations only (no cell sorting). Cell population counts determined using the gating strategy described below.
Gating strategy	Plots of orange fluorescence versus red fluorescence were used to identify and enumerate Synechococcus from other photosynthetic picoeukaryotes, and plots of side scatter versus red fluorescence (with any Synechococcus gated out) were used to enumerate photosynthetic picoeukaryotes. Gates were checked and adjusted manually for every sample to account for variations in fluorescence per cell.

☒ Tick this box to confirm that a figure exemplifying the gating strategy is provided in the Supplementary Information.
